# Dynamics of 1,3-β-D-Glucan in Invasive Candidiasis: A Narrative Review of Microbiological Aspects and Diagnostic Implications

**DOI:** 10.3390/antib15020028

**Published:** 2026-03-27

**Authors:** Maddalena Calvo, Marta Caccamo, Dalila Maria Cammarata, Laura Trovato

**Affiliations:** 1 Laboratory Analysis Unit, University Hospital Policlinico “G. Rodolico-San Marco”, 95123 Catania, Italy; maddalenacalvo@gmail.com; 2 Department of Biomedical and Biotechnological Sciences, University of Catania, 95123 Catania, Italy; caccamomarta@gmail.com (M.C.); dalilacammarata@gmail.com (D.M.C.)

**Keywords:** 1,3-β-D-glucan (BDG), invasive candidiasis, serial BDG monitoring, *Candida albicans*, non-albicans species

## Abstract

Invasive candidiasis (IC) remains a significant cause of morbidity and mortality among critically ill, hematologic, and neonatal patients worldwide. Rapid and accurate diagnosis is essential to guide timely antifungal therapy and improve outcomes. Among available diagnostic tools, 1,3-β-D-glucan (BDG), a polysaccharide component of the fungal cell wall, has emerged as a key biomarker. BDG assays allow for early detection of probable IC, often preceding positive blood cultures, and offer prognostic information based on serial measurements. Species-specific differences in *Candida* cell wall composition influence BDG release and diagnostic sensitivity. *Candida albicans* generally correlates with high BDG levels, whereas *Nakaseomyces glabrata*, *Candida parapsilosis*, and *Candida auris* exhibit variable or lower glucan exposure, limiting assay sensitivity. BDG performance is affected by patient-specific factors, such as prior surgery, transfusions, or coexisting bacterial infections, which may lead to false-positive results. Molecular techniques, including PCR-based assays, provide complementary diagnostic accuracy and species identification, and their combination with BDG testing enhances sensitivity up to 90%. Serial BDG monitoring supports risk stratification and treatment response assessment, with persistent elevations predicting worse outcomes. In neonatal and pediatric populations, optimal cut-off values remain under investigation, highlighting the need for integration with clinical and microbiological data. Overall, BDG represents a valuable adjunct in a multimodal diagnostic workflow, providing both diagnostic and prognostic insights in invasive candidiasis management.

## 1. Introduction

Invasive candidiasis (IC) represents a relevant public health issue, annually accounting for 3.8 million deaths across the world and numerous severe complications among fragile patients [1].

Recent advancements in healthcare facilities caused an increasing invasive fungal infection incidence within intensive care units (ICUs) due to critical patients’ higher life expectations [1]. Skin or mucosal colonization (gastrointestinal tract, urogenital tract) and immunological impairments mainly predispose to *Candida* spp. infections [2]. Specifically, gut microbiome alterations, broad-spectrum antibiotic usage, loss of gut integrity, surgery, and central venous catheter insertion contribute to a transition from commensal to pathogen for this genus [3]. Intensive care unit (ICU) length of stay is also associated with the high risk of *Candida* spp. infections, leading to the development of several “*Candida* scores”, which usually include patient-related risk factors and microbiological surveillance culture results [2]. Otherwise, hematological patients often report *Candida* spp. invasive infections due to extended antifungal treatment as prophylaxis, prolonged neutropenia and corticosteroid usage. Most of those infections are described as “breakthrough invasive fungal infections” (BtIFIs) [4,5].

According to ultimate epidemiological data, invasive fungal infections also include pediatric patients, reporting 1.4 million of annual neonatal deaths worldwide due to fungal neonatal sepsis, pneumonia and meningitidis [3]. Prematurity, immunological impairments, fragile skin barriers, external management devices (central venous catheters, parenteral nutrition), prolonged hospitalization, endotracheal intubation, surgery, and prolonged antibiotic treatment are essential risk factors for severe-infection developments among newborn infants [3]. *Candida* species are responsible for the most commonly encountered invasive fungal infections (IFIs) among hospitalized patients admitted to various clinical settings in high-income countries, describing candidaemia as the most prevalent clinical presentation of IC [6]. Invasive candidiasis among non-neutropenic adults mainly manifests as candidemia and deep-seated candidiasis with or without candidaemia [1].

Recent literature data reported that approximately 1.57 million cases of IC occur annually worldwide, accounting for about half of candidaemia cases. In the United States, the 36% of all-cause in-hospital mortality is associated with candidemia, while in Europe, the 90-day mortality rate approaches 43%, with an attributable mortality rate estimated to be between 22% and 27% [7]. Candidemia mortality rates reach 28–41.6% in Italy, enhancing elevated patients’ management costing according to the Italian National Health Service reports [7]. The recently conducted EUCANDICU study done across nine European countries reported 7.07 episodes of ICU-acquired invasive candidiasis per 1000 ICU admissions, with a crude 30-day mortality of around 42% [8].

**Figure 1:**
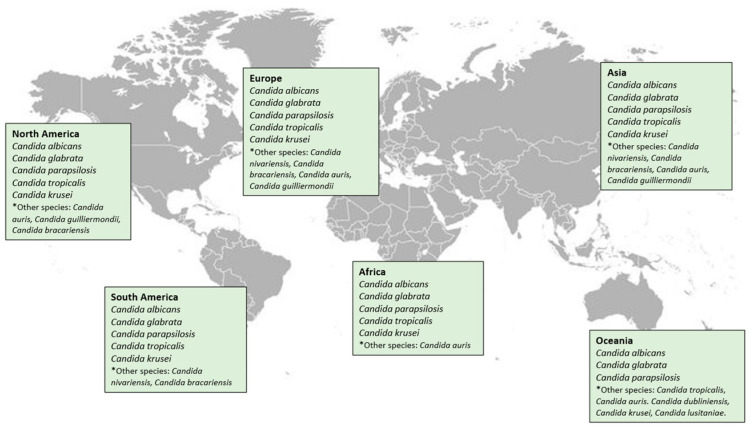
*Candida* species general distribution across the world. This image was entirely created by the authors using online world map database (https://stock.adobe.com, accessed on 28 January 2026).

Invasive across the world, candidiasis reveals significant geographical variation depending on the isolated species. Figure 1 illustrates *Candida* species distribution.

Despite *Candida albicans* accounting for 40–50% of global cases, non-*albicans* species represent an increasing concern due to possible azole resistance. *Candida glabrata* (recently renamed as *Nakaseomyces glabratus*) may occasionally cause clinical challenges due to reported azoles and echinocandin resistance episodes. Another difficult-to-treat isolate is *Candida auris*, an emerging drug-resistant pathogen responsible for several outbreaks in the past decade [9]. It is known to survive on human skin and tough environmental conditions, facilitating rapid transmission in ICU [9]. Finally, a local study analyzed the incidence of *C. albicans* and non-albicans species responsible for invasive candidiasis in Southern Italy between 2020 and 2024. These data showed how *C. albicans* reached the same incidence rate as non-albicans species (*C. parapsilosis*, *C. glabrata*, *C. tropicalis*, *C. krusei*, *C. lusitaniae*, *C. guilliermondii*, *C. famata*, and *Candida nivariensis*) in 2020, showing its supremacy in 2021. The non-albicans isolation rate fluctuated during the following years, reporting percentages higher than the *C. albicans* numbers during 2022 and 2024. Otherwise, 2023 demonstrated a relevant (more than 50%) *C. albicans* percentage [10].

## 2. The 1,3-β-D-Glucan into the Invasive Candidiasis Diagnostic Workflow

### 2.1. Diagnostic Criteria

The European Organization for Research and Treatment of Cancer and the Mycoses Study Group Education and Research Consortium (EORTC/MSG) defined specific criteria for candidiasis diagnostic levels. Proven invasive candidiasis may be defined by positive blood cultures (or other sterile fluids) and/or histopathological evidence of *Candida* spp. fungal elements [11,12]. This classification certainly enhanced the fundamental role of blood cultures as microbiological gold standards within the diagnostic workflow. However, the same criteria define probable invasive candidiasis in the case of positive 1,3-β-D-glucan results (>80 pg/mL) in at least 2 consecutive serum samples. On this premise, this serological marker significantly supports fungal infection diagnosis [11,12].

The 1,3-β-D-glucan (BDG) is a fungal cell wall polysaccharide forming a structural fibrillar network for mechanical strength and integrity. It functions as a pathogen-associated molecular pattern (PAMP), interacting with host immune receptors such as dectin-1 for immune response activation during infectious processes [13,14,15]. BDG released during fungal growth may be measured through several diagnostic assays, allowing for probable invasive candidiasis diagnosis.

### 2.2. Commercial Systems for 1,3-β-D-Glucan Detection

Commercial systems include Fungitell^®^ (Associates of Cape Cod, 124 Bernard E. Saint Jean Drive East Falmouth, MA, USA), Glucatell^®^ (Associates of Cape Cod, 124 Bernard E. Saint Jean Drive East Falmouth, MA, USA), Wako pure chemical assay (FUJIFILM Wako Pure Chemical Corporation, Osaka, Japan), Fungitec-G^®^ (Seikagaku Kogyo Corporation, Tokyo, Japan), and Dynamiker^®^ Fungus (Dynamiker Biotechnology, Tianjin, China). Fungitell^®^ (approved for diagnostic use) and Glucatell^®^ (for research use only) are based on the activation of Factor G in *Limulus* amebocyte lysate, which triggers a protease cascade resulting in a measurable optical change proportional to BDG concentration [14]. Otherwise, Fungitec-G provides colorimetric assays based on the activation of Factor G in *Tachypleus tridentatus*, and the Wako test includes a spectrophotometric rection. Reported diagnostic sensitivity and specificity vary by assay and patient population, generally demonstrating moderate to high negative predictive value and variable positive predictive utility depending on cut-offs and clinical context [13,14]. Table 1 summarizes the main commercial assays’ characteristics, reporting methodologies and cut-off concentrations.

### 2.3. 1,3-β-D-Glucan Diagnostic Accuracy

Regarding non-hematological adult patients, BDG levels are able to discern between patients effectively diagnosed with IC, reflecting elevated values several days before a positive culture. An interesting finding is the frequency of positive BDG early in the IC admission and the subsequent decrease in these levels [15]. It is unknown whether these represent subclinical infection early in the ICU admission or whether this is related to iatrogenic causes such as translocation/leaching, or introduction of BDG into the bloodstream [15]. Some studies demonstrated a negative predictive value higher than 90% and a positive predictive value higher than 70% for BDG usage among ICU patients, recommending two consecutive positive dosages to increase the positive predictive value [15]. Moreover, clinical strategies often integrate the *Candida albicans* germ tube antibody (CAGTA) to enhance the BDG performance in diagnosing IC [16,17]. Patients with hematologic malignancies are a frequent target for BDG dosages. Scientific literature documented a sensitivity of 61% and a specificity of 91% for these patients. However, this condition slightly changes in the case of two consecutive positive results, reaching a specificity of 99%. Otherwise, the prognostic value is difficult to establish due to the long serum half-life and the persistence of high values in these patients. Official guidelines recommend serial BDG measurements (once of twice per week), improving the positive predictive value (PPV) [18].

Invasive fungal infection (IFI) development is more frequent among patients with previous invasive viral infection. For instance, COVID-19 patients are extremely predisposed to IFI with mortality rates reaching 48.5%. A study hypothesized a correlation between BDG prognostic value in those patients, discovering a statistically significant correlation (*p* = 0.0026) between BDG concentration higher than 31 pg/mL, rising age, and mortality [19,20].

Respiratory or gastrointestinal inflammation during the COVID-19 disease probably increases the gut permeability, determining the colonizing fungi bloodstream translocation and consequent fungemia episodes [19,20]. The above-mentioned evidence indicates that serum BDG both supports diagnosis and carries prognostic information. Persistent high concentrations of BDG have been associated with worse clinical outcomes and increasing mortality, while serial declines of the same markers correlate with better survival. These assumptions highlight BDG’s utility for monitoring treatment response and risk stratification [20].

Regarding different patients’ categories, non-specific BDG serum detection may be related to surgical gauzes (laparoscopic or open surgery); albumin, plasma or immunoglobulin transfusions; haemodialysis; and usage of intravenous antimicrobial or antineoplastic drugs, including glucans (sizofiran, lentinan) [15].

Moreover, Gram-negative bacteremia episodes may enhance BDG levels due to the presence of periplasmic glucans within their cell structure. Finally, non-glucan-free laboratory equipment has a negative impact on BDG specificity, frequently causing contamination and false positive results [15]. Serum BDG is increasingly explored as a biomarker to support the diagnosis of invasive fungal disease in neonates, children, and adolescents. However, current evidence is limited and interpretations remain uncertain due to the insufficient accuracy of conventional cut-off values. Some studies hypothesized standardizing cut-off values higher than 300 pg/mL, especially in acute post-transplant phases for pediatric hematological patients [21,22,23,24]. Consequently, results must be integrated with future clinical and microbiological data [21]. Finally, the negative predictive value may exclude invasive fungal infections in adult and children, but insufficient evidence is documented for its use in neonates [22,23,24,25,26]. The scientific literature reported statistically significant differences in BDG sensitivity depending on the identified *Candida* species, particularly lacking for *Candida parapsilosis* and *Candida auris* [22,23,24,25,26]. Herein, we propose a comprehensive review summarizing this interesting microbiological aspect and describing BDG trends in the case of different common or uncommon *Candida* species.

The final purpose of the manuscript is to emphasize the clinical impact of such differences within critical patients’ management.

## 3. 1,3-β-D-Glucan and Different *Candida* Species

Several comparative analyses reveal that the cell wall glucan architecture markedly varies among *Candida* species. Species such as *Candida albicans* exhibit a dynamic modulation of β-glucan exposure, whereas non-*albicans Candida* often display more structurally constrained glucan matrixes. These compositional divergences are further linked to differential immune recognition, as variations in glucan presentation modulate host–pathogen interactions [25,26,27,28,29,30,31,32,33,34,35,36,37,38,39].

### 3.1. Candida albicans

The World Health Organization (WHO) included *C. albicans* within the fungal pathogen critical group due to its virulence and spread among invasive fungal infections’ aetiologies [25]. This species has an outer layer of mannoproteins and an inner polysaccharide layer composed of chitin (2–10%) and β-glucans (60%) as 1,3-β-D-glucan and 1,6-β-D-glucan within its cell wall. Despite its crucial structural role, 1,6-β-D-glucan function and synthesis during fungal growth partially remains underexplored. Otherwise, definitive information is available on 1,3-β-D-glucan and its immunological stimulation during invasive infections. According to this assumption, *C. albicans* tends to mask this antigen though the mannoproteins layer to evade the immunological response of human hosts. The masking episodes delay phagocytosis and cytokine production [25]. A prospective observational study examined the BDG kinetics at the beginning of a candidaemia episode, during the same episode, and at the end of it. The BDG detection tested positive in 68.2% of the cases, showing a sensitivity of 82.9% [26].

A retrospective study from the Asan Medical Center (Seul, Republic of Korea) demonstrated that *C. albicans* may be associated with statistical significance to positive BDG values in the case of candidemia [27].

### 3.2. Candida glabrata

*Candida glabrata* (currently *Nakaseomyces glabratus*) has a limited glucan quantity within the cell wall due to a major proportion of mannoproteins in the same structure. Mannoproteins contribute to BDG masking during fungal elements’ development, complicating its recognition through human host receptors. Consequently, the mannoproteins/glucans ratio appears to be higher than that of *C. albicans*. This feature impacts on BDG availability and release during infection phases [29]. A recent systematic review and meta-analysis showed that the BDG sensitivity for *Candida glabrata* candidaemia was approximately 74%, considering the 80 pg/mL cut-off [29]. According to previously published studies, this value suggests a moderate sensitivity. However, serial BDG measurements may improve diagnostic accuracy, because *C. glabrata* does not appear to be uniquely associated with high or low BDG levels compared to other non-*albicans* species [29]. Clinicians should interpret negative BDG results with caution, especially in high-risk patients with suspected *C. glabrata* invasive candidiasis, and consider repeat testing or alternative diagnostics when clinical suspicion remains high [29].

### 3.3. Candida parapsilosis

*Candida parapsilosis* expresses lower mannoproteins and higher superficial exposure of BDG and chitin than *C. albicans*. These characteristics lead to increased immunological recognition for human host receptors [30]. The diagnostic sensitivity of serum 1,3-β-D-glucan (BDG) for invasive candidiasis caused by *Candida parapsilosis* is consistently lower than that reported for other major *Candida* species, reflecting species-specific differences in cell wall composition and glucan release during bloodstream infection [31]. A recent systematic review and meta-analysis demonstrated sensitivity values of 61–63% at commonly used cut-off values around 80 pg/mL, significantly lower than that observed for *Candida albicans* and *Nakaseomyces glabratus* [32].

This reduced sensitivity has been attributed to lower circulating BDG concentrations, which are thought to result from reduced 1,3-β-glucan exposure or release from the fungal cell wall during infection [30,31,32]. The issue appears particularly pronounced in critically ill or catheter-associated infections, where fungal burden and host factors may further limit BDG detectability [32]. Consequently, a negative BDG result does not reliably exclude invasive infection due to *C. parapsilosis*, and reliance on BDG testing alone may delay diagnosis in this setting [32,33,34]. These findings support current recommendations that BDG should be interpreted cautiously and used only as part of a multimodal diagnostic strategy when *C. parapsilosis* infection is suspected [32,33].

### 3.4. Candida krusei

The *Candida krusei* cell wall contains a higher chitin content than most other *Candida* species, along with lower mannan compared to *C. albicans* and *C. tropicalis* [34,35]. The reduced mannan level in *C. krusei* exposes the underlying 1,3-β-D-glucan, resulting in its greater potential for release and detection in the bloodstream [36].

Furthermore, preliminary studies investigated mannan structures and glucan exposure and recognition modulation during *C. krusei* invasive infections [37]. The performance of serum 1,3-β-D-glucan (BDG) in diagnosing invasive candidiasis due to *C. krusei* (now *Pichia kudriavzevii*) has been assessed in meta-analytic evaluations of species-specific sensitivity, demonstrating that *C. krusei* consistently exhibits among the highest sensitivities (76%) relative to non-albicans species [30].

The elevated BDG levels and sensitivity reflect *C. krusei* cell wall structure and glucan content, which may favour abundant release of 1,3-β-D-glucan into circulation during bloodstream infection [30]. The relatively high sensitivity supports the integration of BDG results into diagnostic pathways in settings where this species is prevalent. However, BDG should still be combined with culture and antigen/molecular methods to maximize diagnostic accuracy [30].

### 3.5. Candida tropicalis

*Candida tropicalis* has a cell wall architecture similar to *C. albicans* in terms of carbohydrate composition, with chitin levels being moderately high but not as elevated as in *C. krusei* [34,35]. The mannan content in *C. tropicalis* is also similar to that of *C. albicans*, with extensive terminal mannose chains that mask the inner β glucan layer, reducing direct exposure of 1,3-β-D-glucan on the cell surface [34,35]. The combination of abundant mannoproteins and moderately proportioned glucan results in intermediate exposure and release of BDG compared to *C. krusei* and species with lower glucan exposure [30]. As a result, pooled BDG sensitivity for *C. tropicalis* candidemia tends to be moderate (approximately 70%), consistent with its structural characteristics that partially conceal 1,3-β-D-glucan relative to *C. krusei* [30]. These findings suggest that *C. tropicalis* releases sufficient 1,3-β-D-glucan into the bloodstream during invasive infection to be detected with moderate sensitivity by BDG assays, reflecting cell wall glucan exposure and release kinetics that are intermediate when compared to highly sensitive species and those with reduced BDG detectability [30]. While species level clinical studies focusing purely on *C. tropicalis* remain limited, the meta-analytic data support the notion that BDG measurement contributes meaningful diagnostic information for suspected *C. tropicalis* candidaemia, especially in high-risk patient populations [30].

### 3.6. Candida auris

The *Candida auris* cell wall exhibits a unique architectural organization that distinguishes it from other Candida species. Its multilayered structure is primarily composed of an inner core of chitin and 1,3-β-D-glucan, covalently linked to an outer layer of highly mannosylated proteins [38]. On one hand, the above-mentioned structure contributes to environmental resilience and resistance to echinocandins [39]. On the other hand, the thick external mannoproteins layer and the complex glucan reticular structure disable the conventional BDG “sloughing”, limiting its dispersion into the bloodstream [39,40,41]. Regarding BDG diagnostic detection, *C. auris* releases limited antigen quantity, demonstrating median levels next to the 80 pg/mL cut-off (50–60 pg/mL), or slightly higher than this value. This evidence impacts the diagnostic sensitivity (40–60%) [39,40,41,42,43,44,45,46,47,48].

### 3.7. Candida guilliermondii

*Candida guilliermondii* correlates to systemic candidiasis in 2–5% of the case, reporting a mortality rate of 27–49%. According to experimental studies, *C. guilliermondii* shows higher mannan (47.8 ± 4.0%) and phosphomannan (131.2 ± 8.9 µg) levels than other *Candida* species. On the other hand, glucan (50.0 ± 5.0%) and chitin (2.2 ± 1.0%) reach lower percentages. As a consequence, the BDG level does not significantly impact the serum diagnostic sensitivity [49].

On the basis of a retrospective case–control study from the University of Freiburg, BDG median levels were 88 pg/mL for Fungitell assay and 5.9 pg/mL for the Wako β-glucan test in the case of proven *C. guilliermondii* candidiasis [32]. Furthermore, other published studies indicated completely negative BDG values during the same clinical and microbiological conditions [47,48,49,50].

### 3.8. Candida (Nakaseomyces) nivariensis and Candida (Nakaseomyces) bracariensis

*Candida (Nakaseomyces) nivariensis*, *Candida (Nakaseomyces) bracarensis* and *Candida glabrata* sensu stricto are included within the *Candida glabrata* sensu lato complex [34]. *Candida nivariensis* was described in 2005 after its isolation from Spanish and English patients, who probably acquired this yeast from the natural environment surrounding the hospital setting [35]. The described isolates demonstrated high azole resistance rates, corresponding to several *C. glabrata* subsets [35]. Additionally, *C. nivariensis* strains reported elevated echinocandin susceptibility due to a high glucan percentage within the cell wall. Although published data are very limited, one case report documented a *C. nivariensis* catheter-related fungemia with high BDG levels (153.7 µg/L) [36,37,38]. Moreover, one research project published interesting results about *C. nivariensis* identification among a large *Candida* spp. Italian collection [51]. *Nakaseomyces*/*Candida bracarensis* was firstly identified in healthcare settings in Portugal and the United Kingdom, demonstrating high susceptibility to echinocandins. This susceptibility trend was hypothetically linked to high glucan percentage within the cell wall, but insufficient studies have been conducted on the diagnostic role of BDG in the case of this *Candida* species [36,39].

### 3.9. Other Candida Species

Rare *Candida* species such as *Candida famata*, *Candida lusitaniae*, and *Candida rugosa* rarely cause invasive fungal infections. Few studies correlated their isolation to specific BDG values, producing only general data about all the uncommon *Candida* species. According to those data, BDG levels demonstrated low median values (79 pg/mL), suggesting a scarce glucan release during bloodstream invasion [30].

Table 2 summarizes the different BDG sensitivities for the cited *Candida* species, using the generic “other *Candida* species” label for *Candida lusitaniae*, *Candida dubliniensis*, *Candida kefyr*, and *Candida guilliermondii*. These above-mentioned species did not furnish sufficient data about BDG sensitivity [6].

## 4. Comparison Between 1,3-β-D-Glucan and Other Diagnostic Methodologies

Despite low sensitivity (38–50%) and prolonged turnaround time, blood cultures remain the gold standard in diagnosing invasive candidiasis. Meta-analytic data reported a global sensitivity and specificity of 80–81% for BDG combined with culture-based methods, markedly outperforming culture alone [52,53]. The BDG assay has shown substantially higher sensitivity than cultures, detecting *Candida* in both candidaemia and deep-seated infections along with other specific biomarkers such as CAGTA [54,55,56]. Regarding molecular techniques, polymerase chain reaction assays (PCR-based assays) reported higher sensitivity than BDG dosages, along with comparable specificity. Both BDG and PCR assays exceeded culture in terms of reproducibility and sensitivity [57]. However, PCR performance is heterogeneous depending on target choice, specimen type, assay standardization, and dedicated expertise [58]. Moreover, some reports showed a higher BDG sensitivity than molecular methods in candidaemia cohorts, documenting its earlier positivity without any correlation to sampling time or culture positivization [58]. Studies combining BDG and PCR reported a 90% sensitivity, overcoming all the individual test limitations [29,59]. PCR methods are not currently included within the official diagnostic algorithms, but scientific evidence suggests that BDG, culture and molecular diagnostics may constitute a multimodal workflow for early candidaemia detection and prompt antifungal stewardship [29,54,55,56,57,58,59].

## 5. Discussion

BDG release and laboratory dosages are heavily influenced by microbiological differences within the diverse *Candida* species cell wall. Particularly, this manuscript emphasized how thick mannoprotein layers may protect glucans’ exhibition in *C. albicans* and *C. glabrata*, impacting their release into the bloodstream. However, *C. albicans* correlates with high BDG concentrations during invasive candidiasis episodes, supporting microbiological diagnosis [30]. In our opinion, this aspect underlines fundamental information due to frequent *C. albicans* isolation in the case of severe fungal infections. Moreover, several authors discussed that *C. parapsilosis* and *C. auris* reveal scarce quantities of glucans within the cell wall, demonstrating limited sensitivity during laboratory investigations [30]. The above-mentioned species-specific considerations support the impossibility of excluding IC in the case of negative BDG among high-risk patients.

Despite variable sensitivity rates depending on the isolated *Candida* species, BDG dosage may show higher predictive positive values after serial serum measurements. Consecutive dosages are recommended in most fragile patients to better investigate eventual false positive results [18,60]. According to several Italian studies, clinical microbiology laboratory should monitor the BDG trend among IC patients. These data demonstrated how the mortality rates decrease along with BDG serum concentration in patients harbouring IC risk factors. Otherwise, persistent high measurements predict critical outcomes, highlighting the association between this serological marker and fungal disease severity [57,61,62]. Unfortunately, persistent positive results should always be investigated, considering all the possible mystifying variables (iatrogenic or environmental glucan sources) [54,55,56]. Furthermore, BDG is often influenced by comorbidities (COVID-19, sepsis), which contributes to its persistence in the bloodstream, along with the marker’s long half-life and variable clearance depending on the analyzed patient [19,20]. On these premises, the BDG dosage should always be included within a wider diagnostic protocol including culture-based methods and extended clinical evaluations of symptoms or risk factors. BDG measurements demonstrated comparable or inferior sensitivity to PCR assays, not providing species-level identification. However, several studies demonstrated that this marker may become positive earlier than both PCR and culture assays, enhancing its potential synergistic effect in combination with other diagnostic procedures [29,58,59,63]. Previously published papers emphasized the importance of combining BDG and other laboratory markers, underlying different sensitivity rates depending on the involved *Candida* species [64]. Beyond its diagnostic role, BDG also revealed prognostic potential in serial declines, which have been associated with improved survival. According to this information, we would like to highlight the importance of continuously measuring BDG to improve antifungal treatment monitoring and risk stratification for critical patients.

## 6. Conclusions

In conclusion, serum 1,3-β-D-glucan is a valuable biomarker for the early detection and monitoring of invasive candidiasis across diverse patient populations. Its diagnostic performance varies according to *Candida* species, with *C. albicans* showing the highest detectability and *C. auris* or *C. parapsilosis* demonstrating lower sensitivity. Serial BDG measurements may improve predictive accuracy and prognostic value, highlighting trends associated with treatment response. Integration with blood cultures and molecular assays enhances the overall diagnostic reliability of 1,3-β-D-glucan measurements. Ultimately, BDG represents an interesting component of a multimodal diagnostic workflow, supporting timely antifungal therapy and better patient managements.

## Figures and Tables

**Table 1:** Summary of the existing commercial assays detecting BDG from serum samples.

Assay	Sensitivity	Specificity	Method	Cut-Off
Fungitell^®^ (Associates of Cape Cod, 124 Bernard E. Saint Jean Drive East Falmouth, MA, USA)	27–100%	0–100%	Colorimetric assay	>80 pg/mL
Glucatell^®^ (Associates of Cape Cod, 124 Bernard E. Saint Jean Drive East Falmouth, MA, USA)	50–92%	41–94%	Colorimetric assay	>80 pg/mL
Wako pure chemical assay (FUJIFILM Wako Pure Chemical Corporation, Osaka, Japan)	67–88%	60–85%	Colorimetric assay	>20 pg/mL
Fungitec-G^®^ (Seikagaku Kogyo Corporation, Tokyo, Japan)	50–86%	89–100%	Turbidimetric assay	>11 pg/mL
Dynamiker^®^ Fungus (Dynamiker Biotechnology, Tianjin, China)	64–81%	78–80%	Chemiluminescence or spectrophometric assay	>95 pg/mL

**Table 2:** Summary of the different BDG sensitivities depending on the isolated *Candida* species, labeling *C. lusitaniae*, *C. dubliniensis*, *C. kefyr*, and *C. guilliermondii* as “other *Candida* species” [6].

*Candida* Species	Medium BDG Value (pg/mL)	BDG Sensitivity Value (%)
*C. albicans*	345	73
*C. glabrata*	356	74
*C. parapsilosis*	95	63
*C. tropicalis*	324	70
*C. krusei*	417	76
*C. auris*	62	51
Other *Candida* species	79	44

## Data Availability

No new data were created or analyzed in this study. Data sharing is not applicable to this article.
